# Has Culling Been Properly Assessed as a Valid and Justified Control Intervention Measure for Zoonotic Diseases?

**DOI:** 10.1371/journal.pntd.0000541

**Published:** 2009-10-27

**Authors:** Maria Vang Johansen, Mary-Lou Penrith

**Affiliations:** 1 DBL - Centre for Health Research and Development, Department of Veterinary Disease Biology, Faculty of Life Sciences, University of Copenhagen, Copenhagen, Denmark; 2 Department of Veterinary Tropical Diseases, University of Pretoria, Pretoria, South Africa; University of Oklahoma Health Sciences Center, United States of America

In their *PLoS Neglected Tropical Diseases* research article “A Pilot Study for Control of Hyperendemic Cystic Hydatid Disease in China,” Zhang et al. [1] describe a research project conducted from 1987 to 1994 in Xinjiang, a multiethnic and multireligious province in western China. The study aimed to assess the efficacy and cost-effectiveness of applying monthly praziquantel treatment to dogs on the prevalence of hydatid disease in sheep. As a part of the study a large number of dogs were caught and killed. The approach described in the paper leaves the reader with a number of dilemmas regarding ethics, validity of the research, and research ethical questions.

Culling animals has been used in many parts of the world as a highly effective way to control and eliminate various infectious diseases of both veterinary and human health importance [2]. Enforcement has been a necessary component as many animal owners do not necessarily agree to this utilitarian approach at the expense of their animal, especially if the animal is not apparently ill or suffering. Regardless of enforcement, if market-level compensation is not paid to owners of animals compulsorily slaughtered, illegal movements may occur and the disease can spread faster and last longer as a result [3]. It is therefore also very clearly stated in the *FAO manual on procedures for disease eradication by stamping out* [2] that although often culling is the most cost-effective strategy, several social, economic, and other factors need to be evaluated before stamping out can be selected. These factors include “whether or not slaughter of infected animals is likely to gain community acceptance on religious, ethnic, animal welfare and other social and economic grounds” [2]. The fact that the impact of the stamping-out approach on livestock and companion animal owners goes far beyond financial loss is often overlooked [4].

In order to justify culling of animals as part of a control strategy, substantial evidence for its necessity is needed. Making evidence-based decisions requires, first and foremost, valid and justified research, which is essential to obtain the best available approximation to the truth [5] and requires that the outcome can be assessed objectively, that the data obtained can be generalized, and that the research can be reproduced. Strict categorization of test and outcome variables is essential to assess any effect. Thus to assess the effect of culling will require a study design in which animals are randomly allocated into cases and controls, whereas biased culling of haphazardly caught animals cannot be regarded as valid research. For valid research to be justified, it must be conducted in a way that respects and protects, and it must use relevant subjects who share risks and benefits without bias, as described in the International Ethical Guidelines for Biomedical Research Involving Human Subjects [6]. Since preventive measures for zoonotic diseases are made to safeguard humans rather than the animals, there is a strong argument for the justified research approach to evaluate them. Research assessing effectiveness of zoonotic disease control programmes must also adhere to these principles, and each measure should be assessed independently in a justified manner. Before introducing culling as a component in a control programme for zoonotic (or other) diseases, a risk analysis should be undertaken that considers the different options [7]. This should include a comprehensive assessment of the impact and acceptability of the proposed measures in the target communities. Application of universal standard recommendations is not a viable option, as cultural and religious beliefs differ throughout the world and call for local adaptations.
